# A review of recent advances in generative artificial intelligence models for biomolecular sciences

**DOI:** 10.1016/j.apsb.2025.12.012

**Published:** 2025-12-11

**Authors:** Jian Jiang, Daixin Li, Guilin Wang, Nicole Hayes, Yazhou Shi, Huahai Qiu, Bengong Zhang, Tianshou Zhou, Guo-Wei Wei

**Affiliations:** a Research Center of Nonlinear Science, School of Mathematical and Physical Sciences, Wuhan Textile University, Wuhan 430200, China; b Department of Mathematics, Michigan State University, East Lansing, MI 48824, USA; c Key Laboratory of Computational Mathematics, Guangdong Province, and School of Mathematics, Sun Yat-sen University, Guangzhou 510006, China; d Department of Electrical and Computer Engineering, Michigan State University, East Lansing, MI 48824, USA; e Department of Biochemistry and Molecular Biology, Michigan State University, East Lansing, MI 48824, USA

**Keywords:** Generative artificial intelligence models, Biomolecules, Drug discovery, Protein engineering, Variational autoencoders, Generative adversarial networks, Diffusion models

## Abstract

Generative artificial intelligence (AI) models, a class of AI techniques that learn data distributions to synthesize novel samples, have emerged as impactful tools across scientific disciplines. In recent years, these models have found extensive applications in fields such as natural language processing and biomedical sciences. Despite their growing influence, comprehensive reviews on the application of generative models in biomolecular sciences remain limited. In this review, we provide a systematic overview of recent advances in generative models applied to biomolecular sciences. We discuss several prominent generative architectures, including variational autoencoders, generative adversarial networks, and diffusion models, highlighting their applications in molecular design and bioinformatics. Additionally, we examine how these models contribute to critical challenges such as molecular property prediction and molecular generation. Finally, we discuss key challenges that remain in this field, including model interpretability, scalability, and the need for high-quality molecular datasets. We highlight emerging research directions that aim to overcome these limitations and propose strategies for improving the reliability and applicability of generative models in biomolecular problems. Through this review, our objective is to provide researchers with a comprehensive understanding of the current landscape of generative modeling in biomolecular sciences and to inspire further advancements in this interdisciplinary area.

## Graphical abstract

This article overviews generative artificial intelligence models, highlighting their key applications in drug discovery, reaction prediction, protein design, and integration with quantum methods, while showcasing various architectures like VAEs, GANs, and diffusion models.

**Figure:**
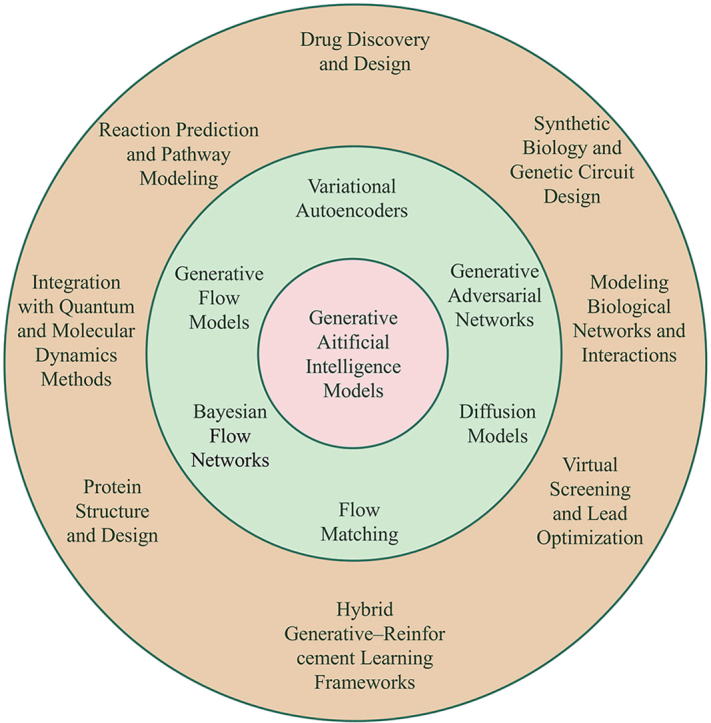

## 1 Introduction

In the past decade, machine learning (ML) has transformed biomolecular sciences, including molecular biology, biochemistry, and structural biology, allowing researchers to address complex biological questions, design novel compounds, and predict molecular properties^1^. Among various ML paradigms, generative artificial intelligence (AI) models stand out for their ability to synthesize new molecular structures and properties from learned representations^2^. This not only accelerates discovery but also enhances creativity in molecular design, reduces experimental time and costs, and facilitates deeper exploration of biomolecular sciences. Generative models learn the data distribution to generate new data similar to real data. By learning the statistical characteristics of the sample data, they can generate new data that mirrors the training set. These models are widely used in biomolecular sciences to accelerate drug discovery and protein engineering, improve experimental efficiency, and reduce development costs^2^^,^^3^. Techniques such as generative adversarial networks (GANs)^4^, variational autoencoders (VAEs)^5^, and diffusion models (DMs)^6^ can help researchers efficiently search and optimize within a vast molecular design space, driving technological advancements in multiple fields^7^.

In biomolecular sciences, generative models are used to predict and design molecular structures and properties, especially in drug design, protein design, and chemical reaction prediction. These models, as a subset of ML, learn from existing data to generate new structures with desired properties by capturing the relationship between structure and function. Unlike other models, generative models focus on sampling data distributions to design molecules with specific properties, saving both time and cost^8^.

The field of generative models is rapidly evolving. Existing reviews on the application of generative models in biomolecular sciences are limited, and further exploration is needed. This paper examines their potential to transform biomolecular sciences by offering innovative solutions and advancing our understanding of molecular systems. Despite challenges such as data scarcity, interpretability, and computational demands, future developments should focus on improving model accuracy, interpretability, and integration with experimental research. We analyze the strengths, limitations, and prospects of generative models, demonstrating how they can accelerate scientific discovery and innovation in biomolecular sciences^9^.

The remainder of this article is organized as follows. Section 2 introduces generative technologies, methods, and theoretical foundations. Sections 2.2–2.3 provide detailed overviews of different generation methods and mathematical foundations. Section 3 discusses the applications of generative models in biomolecular sciences. Section 4 addresses current challenges and limitations, while Section 5 explores future prospects in biomolecular sciences.

## 2 Overview of generative models

### 2.1 Introduction to generative modeling techniques

Model generation is a subfield of ML that focuses on learning data distribution and generating relevant features of the original data^10^. Unlike discriminative models, it focuses on understanding the data generation process and enhancing data by capturing patterns and characteristics. It is derived from statistical methods, similar to Gaussian mixture models and Markov chains^11^. Baum et al.^12^ developed hidden Markov models in the 1960s. These early models paved the way for subsequent generative models to comprehensively evaluate molecular designs.

The core goal of generative modeling is to learn the joint probability distribution p(x) of the data, where x represents the data (*e*.*g*., images, texts, or audios)^13^. By learning this distribution, generative models reveal the underlying structure and generate new samples that preserve statistical properties such as texture, structure, and pattern. In biomolecular fields such as molecular biology, structural biology, and biochemistry, these models are used to learn and generate the distribution and structural properties of complex molecules, aiding in the design of new molecules and accelerating the exploration of chemical space.

Text generation models have been used in various fields, including image generation, drug discovery, protein structure prediction, and protein engineering. Some of the key advantages of generative models are the ability to synthesize new data, which plays an important role in data augmentation, and to enable probabilistic reasoning to enhance the creativity of AI systems^14^.

As generative models continue to demonstrate their vast potential in various fields, particularly in biomolecular sciences, understanding the strengths and limitations of different generative approaches becomes increasingly important. To better evaluate and select the most suitable generative model, the following section will provide a detailed comparison of key models, including VAEs, GANs, DMs. By examining these models, we can gain deeper insight into their performance and challenges in various applications, offering more targeted guidance for real-world use cases.

### 2.2 Comparison of different generative approaches

**Figure 1:**
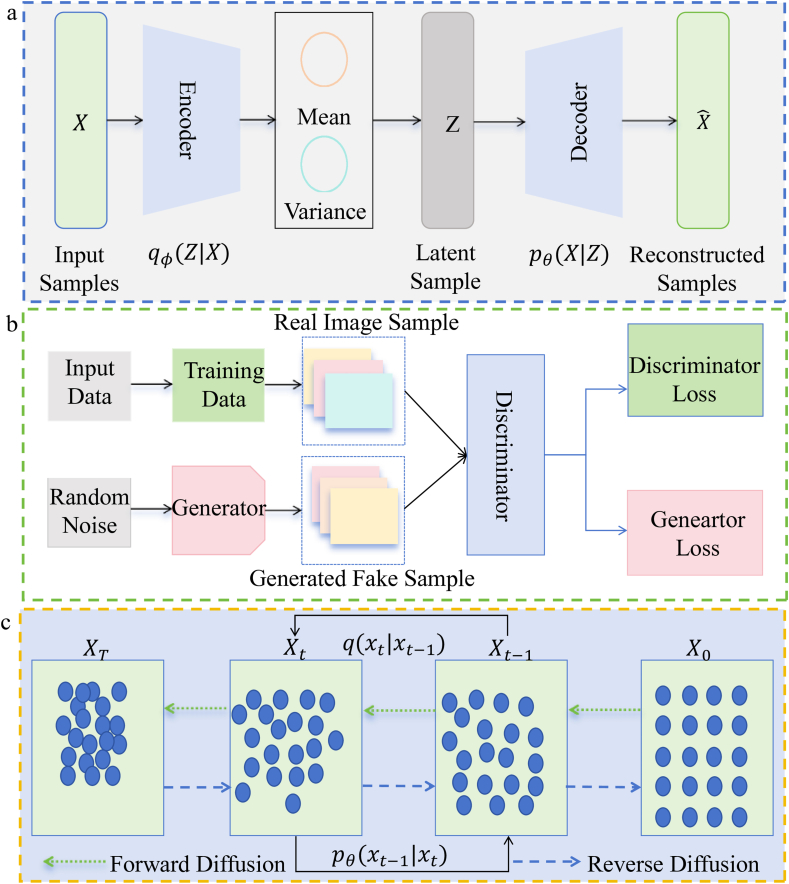
Flowcharts of three different generation methods for generating models. (a) Variational autoencoder extracts the encodings mainly through the encoder. In the hidden space Z, these encodings are treated as latent representations of the latent variables, and then passed to the decoder to produce an output close to the original input data. (b) The process diagram of generative adversarial network uses the generator model and the discriminator model to perform mutual game learning to generate output. (c) The diffusion model includes forward diffusion process and reverse diffusion process. The green line segment represents the forward diffusion. The blue line segment represents the reverse diffusion.

The landscape of generative modeling has evolved, with various techniques excelling in different domains. VAEs, GANs, and DMs are among the most widely adopted methods, and their flowcharts are shown in Fig. 1. Next, we compare these approaches along key dimensions, such as their architecture, training process, strengths, and weaknesses.

VAEs are probabilistic generative models that combine autoencoders with variational inference. As shown in Fig. 1a, the model consists of an encoder, which learns a function $q_{\phi }\left(z|x\right)$ to map input data x to a latent variable z, assumed to follow a Gaussian distribution. The decoder reconstructs the data using a conditional probability $p_{\theta }\left(x|z\right)$. VAEs are advantageous in providing probabilistic interpretation, enabling smooth interpolation in latent space, and effective anomaly detection. However, generated samples often appear blurry, and training can be challenging due to the need to balance reconstruction loss and regularization terms^15^.

GANs consist of two neural networks, a generator and a discriminator, that are trained in a competitive setting. The generator creates synthetic data, while the discriminator distinguishes between real and fake data. Both networks are trained simultaneously in a minimax game, and their specific flowchart is depicted in Fig. 1b. GANs excel at generating high-resolution, realistic images and are widely used in image generation, style transfer, super-resolution, and video synthesis. However, they face challenges such as unstable training, mode collapse, and vanishing gradients. Additionally, their lack of a clear probabilistic structure makes them more complex and difficult to analyze than VAEs^16^.

DMs are generative models that involve forward and reverse diffusion processes. In the forward process, real data $x_{0}$ is progressively corrupted by noise, becoming Gaussian noise through a Markov chain. The reverse process attempts to restore the original data distribution from the noise, with $p_{\theta }\left(x_{t-1}|x_{t}\right)$ representing the conditional probability of the previous state^17^. The detailed flowchart is presented in Fig. 1c. DMs are highly effective at generating high-quality, realistic images, often surpassing GANs in sample quality. Their training process is more stable, avoiding issues like unstable training. However, DMs suffer from slow sampling speed due to multiple refinement steps, which require significant computational resources and time, especially for high-dimensional data like images.

Of course, in addition to the above-mentioned classic generative models, several novel models have emerged, such as flow matching, Bayesian flow networks (BFNs), and generative flow models. Flow matching is a generative approach based on continuous normalizing flows, which directly regresses noise onto the vector field along the data's conditional probability path, avoiding the complex reverse diffusion process and accelerating training and sampling^18^. BFNs, introduced by Graves et al.^19^ in 2023, employ Bayesian inference to update the independent distribution parameters of noise samples and then input them into a neural network to generate new distributions. Unlike DMs, BFNs does not require explicit forward diffusion, and it supports both discrete and continuous data, excelling in image and language tasks. Generative flow models refer to a class of models based on normalizing flows, which use a series of invertible, differentiable transformations to map simple initial distributions (such as standard Gaussian) to complex data distributions, offering precise probability density modeling, expressiveness, and interpretability^20^.

Generative modeling techniques have advanced rapidly, with VAEs, GANs, and DMs demonstrating strong performance in image synthesis, drug design, and molecular generation. Recently, emerging approaches such as flow matching, BFNs, and generative glow models have been introduced, offering advantages like faster training, uncertainty modeling, and exact likelihood estimation. Their applications have also expanded into molecular dynamics (MD) modeling, drug–target interaction prediction, and biomedical data analysis. To facilitate systematic comparison, Table 1 summarizes the advantages and disadvantages of six representative generative models and their main application areas, providing guidance for model selection and application.

**Table 1:** Summary of several commonly used generative models.

Generative model	Advantages	Disadvantages	Main application domains	Ref.
Variational Autoen-coders	- Probabilistic framework with evidence lower bound optimization -Stable training -Continuous latent space	-Samples can be blurry -Difficulty capturing high-frequency details	-Small molecule and protein design -3D molecular Conformation embedding -Text generation and latent semantic Modeling	Wei et al. [15] Zhang et al. [21] Ochiai et al. [22]
Generative adversarial networks	-High-fidelity sample generation -Mature architectures Facilitate stable variants -Smooth latent-space interpolation	-Training instability -Mode collapse risks	-Molecular structure and reaction synthesis -Drug reaction outcome prediction -Text generation and style transfer	Maziarka et al. [23] Zhang et al. [24] Macedo et al. [25]
Diffusion models	-Fine, sharp sample quality -Stable optimization (no adversarial loss) -Flexible conditioning on properties	-Slow sampling due to many steps -3D molecular conformation and drug design	-Molecular graph diffusion modeling -Molecular graph diffusion design -High-quality image generation and restoration	Yang et al. [6] Lin et al. [26] Morehead et al. [27]
Flow matching	-Direct regression of data—noise vector field -Fast training convergence -Adaptive path selection	-Sensitive to path choice -Stability on complex distributions	-Conformer sampling -Molecular dynamics trajectory modeling -Inverse design of small molecules	Lipman et al. [18] Hassan et al. [28]
Bayesian flow Networks	-No explicit forward diffusion required -Support for discrete & continuous data -Unified likelihood ^training^	-Expensive posterior computation -High sampling cost in large spaces	-Uncertainty-aware molecule generation -Bayesian estimation of binding modes -Probabilistic conformer ensembles	Wu et al. [19] Graves et al. [29] Song et al. [30]
Generative flow Models	-Invertible transforms with exact densities -Efficient sampling -High interpretability	-Challenging design of invertible layers -Limited multimodal flexibility	-Molecular density and reaction pathway Modeling -Property-to-structure inverse design in drug discovery -Speech generation and conversion	Zang et al. [31] Bengio et al. [32]

### 2.3 Key theoretical concepts and mathematical foundations

Generative models are grounded in key mathematical concepts from probability theory, optimization, and information theory. These foundations are essential for understanding how generative models work and why some models are more suitable for specific tasks. Next, we discuss the relevant concepts and mathematical foundations.

The first concept is about variational inference and evidence lower bound (ELBO). The goal of these models is to maximize data likelihood, which is often computationally intractable. To address this, VAEs introduce the ELBO as a tractable lower bound to the likelihood. The core objective is to maximize the ELBO, and its formula can be written as:

(1) $$L\left(\theta ,\phi ;x\right)=E_{q_{\phi }\left(z|x\right)}\left[\log\;p_{\theta }\left(x|z\right)\right]-D_{KL}\left[q_{\phi }\left(z|x\right)\|p\left(z\right)\right]$$

where $E_{q\phi }\left(z|x\right)\left[\log\;p_{\theta }\left(x|z\right)\right]$ is the reconstruction loss, in order to measure the decoder's ability to reconstruct data. $D_{KL}\left[q_{\phi }\left(z|x\right)\|p\left(z\right)\right]$ is the regularization term that ensures the latent space distribution approximates a prior, typically Gaussian. VAEs optimize training using variational inference and ELBO, mapping latent space to data space. They generate complex samples and provide training stability, making them a key method in deep generative models^33^.

The next key idea is GANs and the minimax game. GANs are based on an adversarial game between two networks where the generator G and the discriminator D interact. The generator creates realistic data to deceive the discriminator, while the discriminator distinguishes real data from fake. The objective function of GANs can be expressed as:

(2) $$\min_{G}\;\max_{D}V\left(D,G\right)=E_{x∼P_{data}}\left(x\right)\left[\log\;D\left(x\right)\right]+E_{z∼p_{z}\left(z\right)}\left[\log\left(1-D\left(G\left(z\right)\right)\right)\right]$$

in this case, $p_{\text{data}}\left(x\right)$ is the distribution of the real data, and $p_{z}\left(z\right)$ is the distribution of the latent variables. GANs have advanced generative modeling through the adversarial interplay between G and D. The term $E_{x∼P_{data}}\left[\log\;D\left(x\right)\right]$ helps the discriminator better distinguish real data, while $E_{z∼p_{z}\left(z\right)}\left[\log\left(1-D\left(G\left(z\right)\right)\right)\right]$ encourages the generator to improve the quality of generated samples. They are capable of generating highly realistic images and data with wide applications^34^.

The following concept is the diffusion process and reverse sampling. The DMs transform data into noise through the forward process and gradually recover the real data through the reverse process. This process is described using a Markov chain, allowing the generation of data from simple to complex, demonstrating its powerful generative capability^6^.

The mathematical basis of the diffusion process is the stochastic differential equation, which is used to describe the dynamics of the forward and reverse processes^35^. Among them, the forward process is to gradually add noise to the data through the diffusion process, and this process is formulated as:

(3) $$dx_{t}=f\left(x_{t},t\right)dt+g\left(t\right)dW_{t}\text{,}$$

to clarify, $x_{t}$ represents the state of the data at time t, $f\left(x_{t},t\right)$ is the drift term, which dictates the deterministic direction of data evolution, g(t) is the diffusion term, modulating the intensity of the stochastic variation, and $dW_{t}$ corresponds to the increment of standard Brownian motion, capturing the inherent randomness in the process. The reverse process aims to recover data from noise, and its equation is represented as:

(4) $$dx_{t}=\left[f\left(x_{t},t\right)-g\left(t\right)\nabla_{x}\;\log\;p_{t}\left(x_{t}\right)\right]dt+g\left(t\right)dW_{t}\text{,}$$

specifically, $\nabla_{x}\;\log\;p_{t}\left(x_{t}\right)$ is the score function, guiding the denoising process. Through the reverse process, DMs recover data that closely matches the original distribution, making them powerful tools in generative modeling.

The theoretical frameworks of probabilistic inference in VAEs, adversarial optimization in GANs, and noise modeling in DMs form the foundation of generative models. They support the development of more efficient models in fields like image and text generation^36^, and are also transforming applications in biomolecular sciences, particularly drug discovery and protein engineering^2^.

While these theoretical constructs establish the bedrock of generative modeling, their true impact emerges when they are deployed to tackle real-world challenges. In the following section, we turn our attention to the applications of these techniques within biomolecular sciences, examining how VAEs, GANs, and DMs are being used to accelerate drug discovery, guide protein engineering, and drive innovative molecular design.

## 3 Applications in biomolecular sciences

### 3.1 Drug discovery and design: generating novel drug candidates

Drug discovery and design have traditionally been time-consuming and resource-intensive. Generative models, a branch of AI, have the potential to revolutionize this field. Techniques such as VAEs, GANs, and transformer-based architectures can design novel drug candidates with specific properties, including molecule design, property optimization, and target-specific drug discovery. These innovations accelerate and optimize drug development^37^^,^^38^. Moreover, recent perspectives have suggested that generative AI may play an increasingly influential role throughout the drug discovery pipeline^39^.

**Figure 2:**
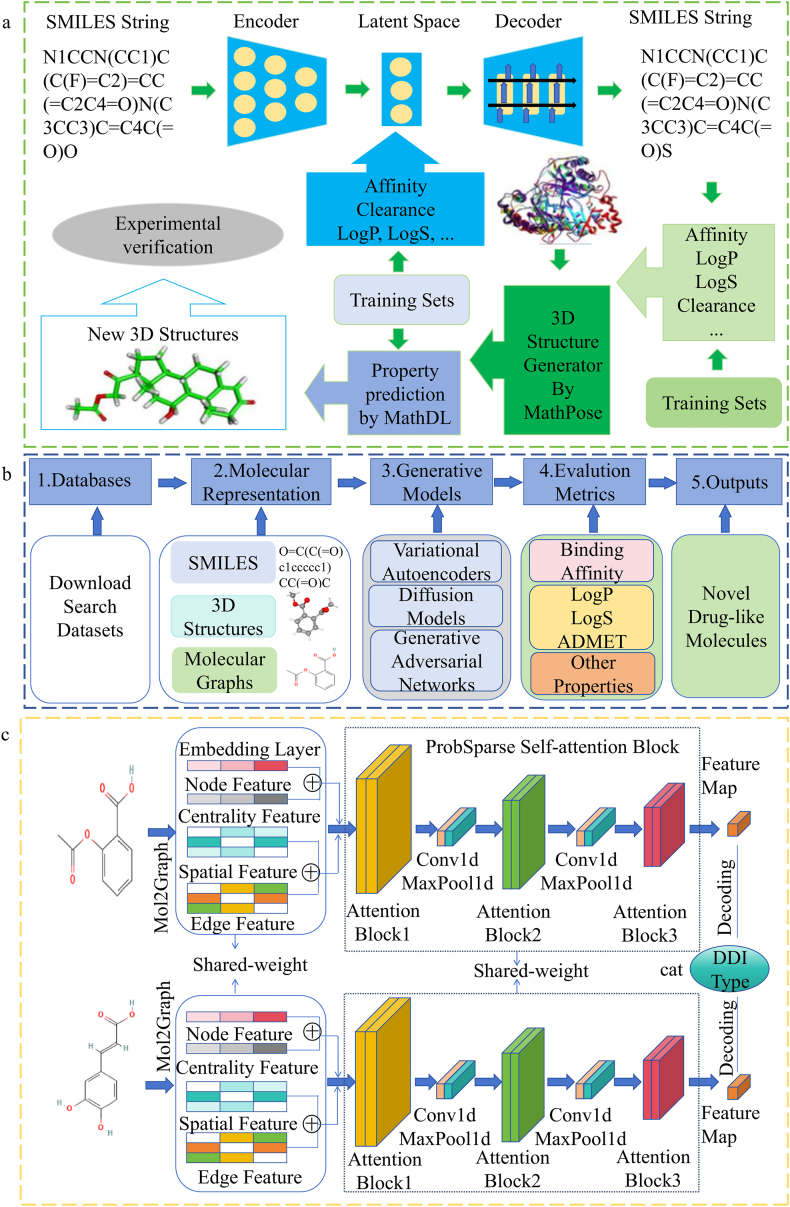
(a) A schematic illustration of a generative network complex. It consists of an autoencoder that takes SMILES strings into a drug-property regulated latent space, a regulated latent space, a LSTM based autodecoder, a multitask network for the evaluation of binding affinity (BA), partition coefficient (LogP), solubility (LogS), clearance, etc., a three-dimensional (3D) structure generator named MathPose, and MathDL^45^^,^^46^, a refined 3D multitask druggable property predictor based on algebraic topology, differential geometry, and graph theory, to select new drug candidate structure^40^. (b) This is an AI-driven drug discovery process diagram^44^. The process begins with extracting relevant information from the collected dataset and representing molecules using SMILES, 3D structures, and molecular graphs. Then, generative models such as variational autoencoders, generative adversarial networks, and diffusion models are employed to generate candidate molecules. These candidates are evaluated based on properties such as BA, log*P*, log*S*, and ADMET (absorption, distribution, metabolism, excretion, and toxicity), ultimately leading to the identification of novel compounds with potential therapeutic value. (c) The overview of Molormer. The original drug graph goes through an embedding layer to form four feature embeddings representing the spatial structure of the drug. These features are processed by two ProbSparse self-attentive blocks, connected and fed to the decoder for final prediction. Drug–Drug Interaction (DDI) refers to the effects that occur when two or more drugs are taken together, potentially altering the effectiveness or side effects of one or both of the drugs^47^.

Scientists have combined generative model-based structure predictions with AlphaFold's capabilities to advance drug discovery. For example, in 2019, Grow et al.^40^ proposed the generative network complex (GNC) platform, which utilized the SMILES autoencoder, deep neural networks, and a three-dimensional (3D) generative network to generate new compounds, predict their properties, and screen drug candidates. The schematic diagram of this approach is presented in Fig. 2a. In 2023, Gao et al.^41^ advanced molecular generation by combining molecular fragments with hierarchical chemical graph representations, employing a multi-resolution graph VAEs for deep molecular generation. Their goal was to generate drug molecules with chemical diversity and potential bioactivity through deep learning. Additionally, in 2023, Wang et al.^42^ integrated deep generative models with stochastic optimization and showcased ChatGPT as a virtual assistant to enhance drug development efficiency. In 2024, Qi et al.^43^ introduced perturbation-conditioned generative model, a perturbation-conditioned deep generative model trained on nearly 100 million high-throughput screening data. It predicted transcriptional responses of 175,549 compounds in 188 cell lines and has demonstrated effective candidate drug screening in multiple metabolic disease models. In 2024, Kanakala et al.^44^ summarized recent advances in generative AI technologies, tools, and practical applications for small molecular drug design. The workflow of drug design is illustrated in Fig. 2b.

Generative models have shown immense potential in drug discovery and design, particularly in accelerating chemical library screening, reducing costs, and exploring new chemical spaces. Despite challenges such as scaffold homogenization, inaccurate ADMET predictions for novel chemical types, conflicts in multi-objective optimization (MOO), target bias, and poor generalization to new scaffolds persist, generative models have made significant progress in generating novel drug molecules and optimizing lead compounds^44^, and are expected to accelerate the discovery of new tracers and optimize their *in vivo* tracking performance and imaging contrast in the field of radio pharmaceuticals and molecular imaging^48^. Looking ahead, improvements in datasets quality, the introduction of explainable AI, and the integration of multi-omics data could provide more comprehensive drug design solutions. Cross-disciplinary research and collaboration will be key to overcoming current limitations, accelerating precision drug development, and driving the revolution of AI-powered drug discovery.

### 3.2 Reaction prediction: learning reaction pathways and outcomes

Chemical reaction prediction is key for designing druggable molecules and macromolecules, advancing drug discovery and synthetic biology. Traditional methods rely on expert rules but struggle with the complexity of the chemical space. Recently, deep learning-based generative models have emerged, with recurrent neural networks, graph neural networks (GNNs)^49^, and transformers driving rapid progress in reaction prediction^50^.

The core task of generative models in reaction prediction is to learn the underlying patterns for generating reaction products. These models understand the language of chemical reactions through large-scale chemical reaction datasets and are able to predict reaction products given the reactants. For example, in 2019, Schwaller et al. developed the molecular transformer, a transformer-based model for reaction prediction. By treating reactions as a sequence-to-sequence problem, this model achieved state-of-the-art accuracy on benchmark datasets^51^. Additionally, in 2024, Jiang et al.^47^ applied the Molorme framework to the transformer model, creating a drug–drug method interaction method that incorporates a lightweight attention mechanism, enhancing performance and reducing computational costs. The general framework is illustrated in Fig. 2c.

Generative models have shown exceptional capabilities in chemical reaction prediction, particularly in biomolecular science, such as enzyme catalysis and drug synthesis pathway design. Their advantages include scalability, flexibility (*e*.*g*., GNNs for molecular graph modeling), strong generalization, and simplified prediction for non-experts. However, challenges remain in generating reaction pathways, including difficulties in capturing transition state information, prediction biases due to sensitivity to substrate stereochemistry, the tendency to produce unrealistic outcomes in multi-component complex reaction systems, and the inability to accurately model dynamic changes at catalyst active sites. Despite these challenges, generative models are essential in modern chemical reaction prediction. Future research will focus on hybrid models, improving interpretability, and integrating with automated synthesis platforms to accelerate the discovery of new proteins and drugs, driving innovation in biomolecular research and biopharmaceuticals^52^.

### 3.3 Virtual screening and optimization of lead compounds

Virtual screening and lead compound optimization are key steps in drug discovery. In recent years, the introduction of generative models has significantly transformed the identification and optimization of drug candidates. While traditional virtual screening relies on methods such as molecular docking and dynamics simulations to assess binding affinity (BA), generative models enable the creation of novel molecules with desirable properties, providing a more efficient and diverse approach^53^.

**Figure 3:**
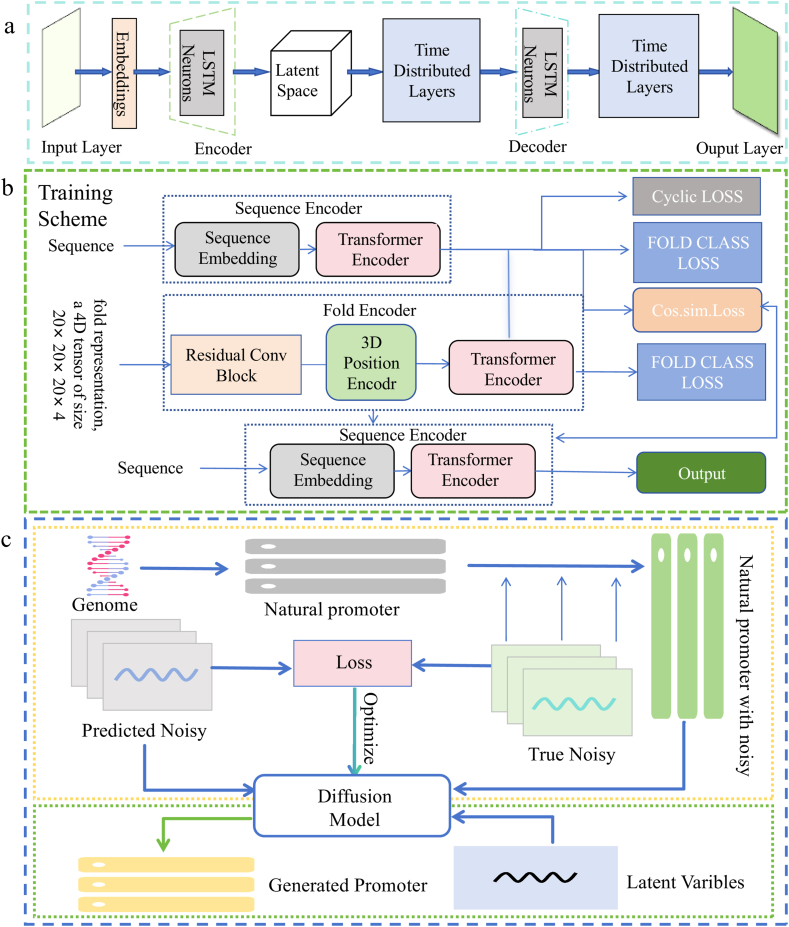
(a) The architecture makes use of embeddings to encode the characters within the text data. It employs a long short-term memory (LSTM) encoder to process the embedded data and map it to a latent space. Additionally, it uses an LSTM decoder to reconstruct the original text data from the latent space representation^57^. (b) The training framework of the Fold2Seq model comprises three key components: the Sequence Encoder, the Fold Encoder, and the Sequence Decoder. The Sequence Encoder transforms the input sequence data into a high-dimensional representation. The Fold Encoder processes this sequence through folding to capture its structural information. Finally, the Sequence Decoder generates the output sequence based on the encoded representation. Together, these components optimize the model's sequence generation capability^59^. (c) Workflow for designing synthetic promoters using a diffusion model. The yellow box represents the training phase. In this phase, random noise is progressively introduced into the natural promoter sequence until the sequence expression matrix is entirely transformed into a random matrix. The diffusion model is employed at each step to predict the noise added, based on natural promoters with noise, thereby facilitating the gradual generation of noise. The green box represents the generation phase. In this phase, latent variables with the same dimensions as the natural promoters are input. The trained diffusion model then denoises these inputs to generate synthetic promoters^26^.

In generative models, especially those based on deep learning architectures such as VAEs, GANs, and reinforcement learning (RL) methods^54^, there has been an increasing application in virtual screening and lead compound optimization^55^. In 2023, Danel et al.^56^ proposed a method combining generative models with molecular docking to accelerate the discovery of new drug candidates and improve virtual screening efficiency. In 2023, Chakraborty and Hasija explored how deep learning methods, particularly generative models such as VAEs and GANs, can efficiently explore potential drug molecular spaces and provide strong support for the drug discovery process^57^. The structure of this model is depicted in Fig. 3a. In 2024, Lu et al.^58^ proposed DynamicBind, a generative model for predicting protein-ligand complexes. By integrating MD with generative techniques, it effectively captures the flexibility and dynamics of binding conformations.

Generative models in virtual screening and lead optimization efficiently predict the activity of novel compounds, explore chemical spaces, and optimize molecular properties. However, they still face several unique challenges in virtual screening, such as generating molecules that often lack core scaffold innovation and tend to get trapped within known chemical spaces, insufficient prediction accuracy for rare structural active sites, and limited capability to assess risks related to potential off-target activities and metabolic transformation pathways. Although generative models can design molecules with strong biological activity, experimental synthesis remains challenging, and deep learning models incur high computational costs in large-scale screening^60^. Future developments will improve the accuracy of generative models by integrating MD, quantum calculations, and docking techniques, while improving drug generation through diverse datasets that include biological activity and ADMET (absorption, distribution, metabolism, excretion, and toxicity) properties^9^. Incorporating synthetic feasibility and real-time feedback optimization will drive the application of generated compounds in experimental synthesis, accelerating the drug discovery process^61^.

### 3.4 Protein structure prediction and design

Prediction and design of protein structures are crucial for understanding protein function and mechanisms, with applications in drug design, protein engineering, and directed evolution^62^^,^^63^. Generative models have shown great potential in this field, as they can directly generate or optimize 3D protein structures from raw sequences, unlike traditional methods such as homology modeling and MD, which rely on existing data.

In generative models, particularly VAEs, GANs and deep neural networks, have been applied in protein structure prediction and design. These models not only predict the 3D structures of unknown proteins, but also generate entirely new protein molecules and optimize their structures to improve functional performance^62^. In 2021, Cao et al.^59^ proposed Fold2Seq, a transformer-based generative model for the design of protein sequences based on target folds. It jointly learned protein sequences and 3D structures, capturing complex sequence-structure relationships, and showed strong performance in handling low-quality or incomplete input structures. The training scheme framework of this model is demonstrated in Fig. 3b.

Generative models in protein structure prediction offer advantages such as efficiency, diversity, and functionality-driven design. They can identify key factors affecting protein folding and function, generating a variety of protein structures. However, they also face several challenges, including difficulties in modeling transient intermediate conformations along folding pathways, limited accuracy in predicting the effects of post-translational modifications, and difficulty in capturing the conformational dynamics of membrane proteins and large multi-domain proteins. Future research should focus on building comprehensive databases for disease-related and species-specific proteins to enhance data support, and improving protein synthesis practicality for experimental validation. Integrating multi-task learning (*e*.*g*., protein folding, function prediction, and drug design) will improve model accuracy, while incorporating biophysical and chemical principles will further enhance prediction reliability. Generative models have made significant progress in protein structure prediction, showing great potential in drug discovery and bioengineering^2^.

### 3.5 Synthetic biology: designing genetic circuits and pathways

A key task in synthetic biology is to design genetic circuits and metabolic pathways for precise cellular control, with applications in biofuel production, drug development, and environmental sensors. Traditional methods are slow and costly, relying on iterative experimentation. Recently, generative models such as VAEs, GANs, DMs, and RL have been applied to automate the generation of new genetic circuits or pathways, accelerating design and reducing costs, thus advancing synthetic biology towards more efficient solutions^64^.

Generative models can learn from large amounts of known gene sequences and metabolic network data to generate new design solutions. For example, GANs can be used to design new promoter sequences. In 2020, Zhang et al.^24^ proposed HpGAN, a GAN-based model to generate specific sequences, while VAEs optimize key enzymes in metabolic pathways, facilitating biological sequence design and gene circuit optimization. In 2024, Lin et al.^26^ introduced a DMs-based generative method for *de novo* design of synthetic promoter sequences, and the method's flowchart is available in Fig. 3c. This method used a stepwise denoising process to generate and optimize high-dimensional promoter sequences, producing several synthetic promoters with high expression efficiency, which showed superior performance compared to traditional designs in experimental validation.

Generative models in synthetic biology offer efficiency, diversity, and customization by rapidly exploring design options for gene circuits and metabolic pathways. For example, HpGAN and DMs generate efficient promoter sequences using large-scale data^24^. However, several challenges remain, such as the unpredictability of *in vivo* behavior due to context-dependent regulation, and difficulties in modeling nonlinear metabolic fluxes under fluctuating environments or cellular states. Future developments will focus on building comprehensive gene sequence and metabolic pathway databases, integrating data from various species and environments, and combining them with automated platforms to accelerate validation. Improving the model interpretability and incorporating MOO will balance factors such as stability, yield, and tolerance, driving the design of more practical gene circuits and pathways^65^.

### 3.6 Understanding biological networks and interactions

Biological networks, including protein-protein interaction networks, gene regulatory networks, and metabolic pathways, are key to systems biology, helping to understand biological functions and responses to environmental conditions. However, their complexity and dynamic nature present challenges in analysis and modeling. Recently, deep learning-based generative models have shown great potential in modeling networks, predicting interactions, and generating novel hypotheses. These models capture intricate relationships and can generate new network structures, predicting previously undiscovered phenomena, even without comprehensive experimental data, offering powerful tools to advance systems biology research^66^.

**Figure 4:**
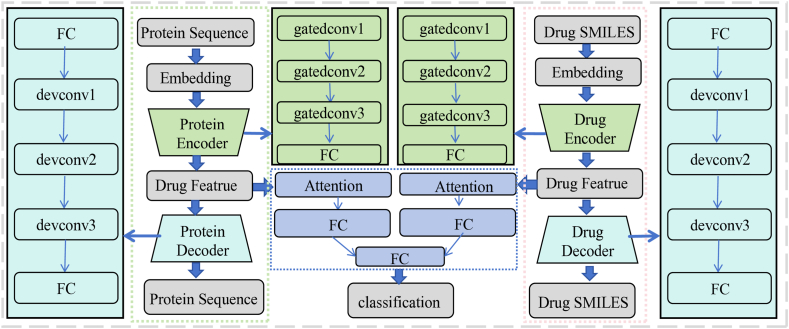
The model comprises three key components: the encoder, the decoder, and the prediction module. Both the encoder and decoder are used to predict drug-protein interactions (DPI). The architecture extracts features from drugs and proteins, applies an attention block to highlight important parts, and passes the output to a fully connected layer for DPI prediction. The feature extraction for drugs and proteins is similar. Text sequences representing drugs and proteins are converted into numerical vectors and then transformed into embedding matrices. The encoding layer contains three Gated Convolutional Neural Networks (GatedCNNs), with Rectified Linear Unit activation after each layer, and a max-pooling layer follows the third GatedCNN to compress the extracted features^21^.

Generative models have increasingly emerged at the forefront of biological network research, with several pivotal studies demonstrating their effectiveness in network prediction and highlighting their potential to discover unknown interactions and generate hypothetical networks. In 2022, Gomari et al.^67^ showed that VAEs can learn nonlinear transferable latent representations from metabolomics data, providing a useful tool for analyzing metabolic networks and disease-related changes. In 2024, Zhang et al.^21^ proposed a VAE-based model with an attention mechanism to predict drug–protein interactions, using deep learning to identify interaction patterns from large-scale drug and protein data, thus accelerating drug discovery and target identification. The schematic diagram of this model is provided in Fig. 4.

Generative models hold great potential in biological network research, enabling faster network design, structural optimization, and prediction of unknown biological relationships, thus reducing experimental burdens. They are particularly useful in drug discovery, precision medicine, and synthetic biology, where they can identify new drug targets, predict drug interactions, customize treatment plans, and design biological networks, accelerating bioengineering advancements. However, their broader application is hindered by challenges such as high uncertainty due to sparse or incomplete interactome maps, and limited capacity to model dynamic, context-dependent network changes. Despite these obstacles, ongoing technological progress will establish generative models as essential tools in systems biology, advancing the study of complex biological interactions and driving breakthroughs in related fields^66^^,^^68^.

### 3.7 The integration of generative models with quantum mechanics and molecular dynamics

The integration of generative models with quantum mechanism and MD holds great potential in biomolecular sciences. They accelerate molecular design and property prediction, reducing the computational cost of quantum calculations, and provide efficient initial structures or conformational evolution pathways for MD simulations^69^. This combination drives molecular discovery, enabling the design of new molecules with specific functionalities.

Quantum mechanism methods play a crucial role in predicting molecular structures and properties, but their high computational costs and complexity limit their application. Generative models can reduce computational costs and improve predictive accuracy through adaptive basis set generation and uncertainty quantification^70^. In 2021, Li et al.^71^ proposed a new quantum GAN mechanism with a hybrid generator and classical discriminator for drug discovery, which supported various number of qubits and quantum circuit layers. Their model with less than 20% of the original parameters can learn molecular distributions as efficiently as its classical counterpart.

In MD simulations, generative models can quickly generate initial conditions and simulate molecular conformation evolution, reducing computational time and enhancing efficiency. They can predict molecular behavior under various conditions and help simulate complex systems. The integration of generative models with quantum mechanics and MD enables efficient conformational sampling but faces domain-specific challenges including potential energy surface inaccuracies and timescale mismatches between MD simulations and generative sampling timescale mismatches between MD simulations and generative sampling^69^.

### 3.8 Development of hybrid models combining generative techniques with reinforcement learning

The integration of generative models and RL offers an efficient solution for molecular design and optimization. Generative models learn chemical space distributions to generate candidate molecules, but struggle to directly optimize target properties^68^. RL, on the other hand, optimizes multi-objective properties through reward mechanisms but faces challenges in exploring high-dimensional and discontinuous spaces. Combining these approaches allows generative models to generate initial molecular structures while RL optimizes target properties, achieving a synergistic effect in molecular generation and optimization^37^, with significant potential in drug design and protein engineering.

For example, in 2021, Hu^72^ applied molecular deep Q-networks to optimize molecular structures for multi-objective problems by treating the molecular construction process as a Markov decision process and using deep Q-learning to guide step-wise modifications. This approach enables the agent to sequentially add atoms or bonds to maximize long-term rewards, demonstrating flexible control over molecule generation. In another study, Koshino et al.^73^ developed a molecular GANs framework that integrates GANs with RL to generate molecules with specific properties. By combining adversarial training with property-based reward signals, this model facilitates goal-directed molecule generation, optimizing features like drug-likeness and synthetic accessibility. Additionally, methods using pretrained generative models and RL mechanisms have shown success in drug design. For example, variational graph autoencoder (VGAE) with Monte Carlo tree search (MCTS), proposed by Iwata et al.^74^ in 2023, combines a VGAE-MCTS to explore the chemical space and optimize molecular properties. The VGAE captures latent molecular representations, while the MCTS navigates the space efficiently through reward-guided search. Similarly, graph-based autoregressive flow, introduced by Han et al.^75^, employs a sequential generation strategy where molecular graphs are built node by node. RL is used to fine-tune the generation process based on the reward of stability and chemical property. Key challenges for hybrid models include discrete action space limitations in molecular graph construction and delayed reward signals in multi-step generation^74^, requiring specialized optimization approaches.

Future research could address these issues through efficient search and optimization strategies, automated reward function design, and collaborative training methods. Incorporating synthetic feasibility prediction models and Pareto-based optimization methods could enhance MOO capabilities^76^. Cross-disciplinary collaboration and quantum-assisted computation could further improve efficiency and applicability in drug discovery and biological science.

### 3.9 Other applications

Although generative models have made significant progress in drug discovery and protein structure prediction, their applications extend far beyond these areas. With ongoing research, generative models demonstrate enormous potential in various biomedical fields, particularly in epigenetics^77^, microbiome modeling^78^, clinical practice^79^, as well as mRNA vaccine design and nucleotide modification optimization^80^.

Generative models are increasingly being applied in biomedicine, showing significant potential in epigenetics, microbiome modeling, clinical practice, as well as mRNA vaccine design and nucleotide modification optimization. In 2025, Mandal et al.77 proposed the supervised GANs method, which effectively combines supervised learning and GANs to apply to labeled genomic or epigenomic data, providing an innovative solution for labeled data analysis in genomics and epigenomics. In 2023, Choi et al.^78^ employed a deep learning method based on GANs to propose DeepMicroGen, a tool to impute missing values in longitudinal microbiome data while preserving the diversity of microbial communities and time-series characteristics. In 2025, Wang et al.^79^ proposed a self-improving generative model that enhances the quality and diversity of generated images. It was successfully applied to clinical decision support, disease prediction, and personalized treatment, providing new solutions for medical imaging and precision medicine. In 2024, Tang et al.^80^ presented the first deep generative framework specifically designed for m1 Ψ-modified mRNAs, termed smart 5 untranslated region (UTR). The model accurately predicted and generated optimized 5′UTR sequences, enhancing mRNA translation efficiency. These advancements highlight the transformative potential of generative models in biomedicine, paving the way for more accurate data analysis, improved medical imaging, and better personalized healthcare solutions. As research continues, the scope of their applications is likely to expand, offering innovative tools for precision medicine and disease management.

Despite the significant potential of generative models in biomedicine, their clinical applications still face some challenges, including the inability to capture patient-specific heterogeneity and inter-individual variability. There is also a risk of generating biologically implausible or unsafe candidates, along with difficulties in designing mechanistic validation and conducting prospective clinical trials. Moreover, ensuring the reliability and accuracy of these models in real-world applications remains a critical challenge.

## 4 Challenges and limitations

### 4.1 Data quality and availability: addressing biases in training datasets

The application of generative models in biomolecular sciences relies on high-quality training data, but such data often suffer from uneven distribution, noise, and biases. The complexity of molecular structures, especially large macromolecules, makes it difficult to standardize molecular features, affecting data quality. Additionally, the limited availability of molecular samples challenges the development of accurate ML models. Researchers often use physical models and theoretical methods (*e*.*g*., molecular mechanics, dynamics, and quantum mechanics) to generate molecular properties and expand datasets^7^. However, these methods have limitations, and biases in simulated data can still affect model accuracy and generalizability. Therefore, ensuring data quality, diversity, and addressing biases are key challenges in applying generative models in biomolecular sciences^68^. Publicly available datasets such as MoleculeNet and therapeutics data commons offer standardized benchmarks and curated molecular data, helping alleviate issues of datasets bias and scarcity. These data sets support tasks such as prediction of molecular properties, estimation of toxicity and modeling of drug–target interaction, making them essential resources for training robust generative models.

Data biases during training can significantly impact model performance and output quality. These biases arise from limitations in training datasets, often dominated by certain molecule categories, restricting the model's ability to generate novel molecules^81^. Strategies like data augmentation, diversity-focused objective functions, and fairness constraints can help improve data diversity and balance. These approaches aim to improve model generalization and improve the quality of generative models in biomolecular sciences^9^.

Balancing innovation and feasibility is crucial, as generated molecules must meet drug design requirements and be experimentally validated. Ensuring the diversity and comprehensiveness of the training datasets is essential for the success of generative models in drug discovery^82^.

### 4.2 Interpretability of generative models in biological contexts

As the interpretability of generative models in ML gains increasing attention, it is crucial to explore how these models can be made understandable, especially in complex fields such as biology^83^. Currently, there is no unified standard, and traditional methods like disentanglement have limitations in practical applications. This highlights the need to evaluate the interpretability of generative models in biological contexts, as understanding the decision-making process can provide valuable insights for applications such as drug discovery.

One method to enhance interpretability is through the use of attention mechanisms, which have been widely applied in natural language processing^84^. In drug discovery, attention mechanisms help identify key features of molecular structures, improving the transparency and interpretability of generative models. Visualization techniques also help improve interpretability, allowing researchers to intuitively understand the decision making process of generative models in drug design^85^, revealing how the model generates drug-like molecules while focusing on key biological concepts during the process.

Integrating generative models with quantum mechanism and MD further enhances interpretability^68^. Several explainable AI frameworks have emerged for generative modeling, such as feature attribution methods and graph explanation tools, which help clarify how molecular features influence generation outcomes^86^. For instance, explainable VAE-based models have been applied in identifying latent variables related to toxicity or solubility, facilitating property-driven molecule design. This integration not only optimizes molecular structures but also helps researchers better understand the decision-making process behind generation, improving transparency and verifiability in drug discovery^87^.

### 4.3 Resource limitation

In recent years, generative models have experienced a leap from hundreds of millions to trillions of parameters (*e*.*g*., OpenAI's GPT-3, GPT-4, Google's Gemini series, DeepSeek's DeepSeek series, etc.), significantly improving generation quality and broadening their range of applications through the expansion of parameter sizes and training data^88^. Beyond traditional text generation, multimodal models for drug design, protein design, and protein structure prediction (such as ChemBERTa, MolGPT, GraphDF, AlphaFold, etc.) showcase the broad applicability of generative technology across various domains. Architectural innovations like transformers, DMs, and GANs have further improved performance, but this comes with increased computational demands and more complex training processes.

Resource demands vary significantly by model type, with VAEs being memory-efficient but requiring extended training periods^89^, while DMs incur high FLOPs per sampling step^90^, creating distinct opti-mization requirements. For example, training GPT-3 requires millions of dollars and spans weeks or even months^91^. In fields like biological and molecular generation, DMs (*e*.*g*., BioDiffusion or MatDiff) necessitate multiple iterations in latent space, resulting in exponential increases in flops requirements, further escalating computational demands. Consequently, the multi-step iteration in DMs not only exacerbates computational burdens but also challenges real-time performance and system response speed. To address this, more efficient optimization strategies and computational architectures are needed to balance performance with resource consumption^90^.

## 5 Future directions

### 5.1 Large language model assisted generation

Rapid advancements in AI are transforming pharmaceutical research. Foundation models, including large language models (LLMs) like GPT-4, can analyze vast literature, propose hypotheses, optimize molecular structures, and support complex decision-making. Their use in drug discovery aims to improve efficiency, reduce costs, and speed up the identification of new therapeutic compounds. Unlike the lengthy and expensive traditional pipeline, AI-driven methods, particularly generative models, offer a data-centric framework. They complement existing computational and experimental approaches and reshape pharmaceutical innovation^92^.

**Figure 5:**
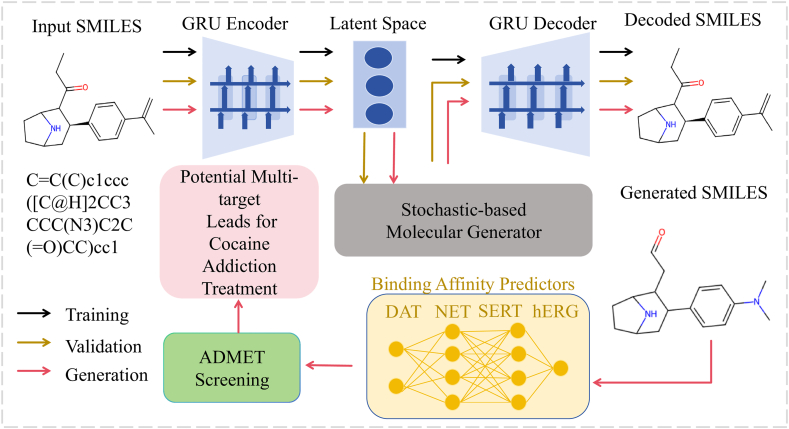
Workflow of the stochastic-based generative network complex (SGNC)^42^. ChatGPT was extensively involved in the building process of the SGNC. Dark arrows show the training process, brown arrows indicate the validation process, and red arrows show the generation process. The SGNC comprises five primary structures: a sequence-to-sequence AutoEncoder in blue, binding affinity predictors in yellow, a stochastic-based molecular generator in grey, ADMET (absorption, distribution, metabolism, excretion, and toxicity) screening *via* ADMETlab 2.0 in green, and potential multiple targets for cocaine addiction treatment in pink.

LLMs, as a subclass of foundation models specialized in processing textual and symbolic information, play an important role in drug development. For example, in 2023, Wang et al.^42^ incorporated GPT-4 into the full drug design workflow. The system facilitated idea generation, methodological analysis, and programming support. They developed a randomly generated network complex using AI-driven molecular design, combined with Langevin equation simulations and DMs analysis. Debugging and optimization were implemented throughout the process. The framework integrated multiple components including a seq2seq autoencoder, affinity prediction models, a Langevin dynamics-based molecule generator, and ADMET screening protocols Ultimately, this approach yielded 15 promising drug candidates for combating cocaine addiction. The workflow for generating a network complex is visualized in Fig. 5.

LLMs show significant potential in drug development, yet challenges persist. Future efforts should focus on integrating LLMs with molecular generation models like DMs and VAEs. RL could be employed to optimize molecular structures, balancing binding scores, biological activity, and synthetic feasibility. Enhancing fact-checking capabilities through cross-validation with authoritative databases (*e*.*g*., PubChem, ChEMBL, AlphaFold) will improve reliability. Robotic lab automation could streamline automated synthesis and testing workflows. Additionally, domain-specific fine-tuning of models may further enhance predictions of drug-likeness, toxicity, and ADMET properties^93^. The case of anti-cocaine addiction drugs shows that LLM-assisted generation can significantly accelerate drug development, but fully autonomous AI-driven drug development still needs further breakthroughs in hybrid modeling, RL and experimental verification.

### 5.2 Generation based on foundation models

By learning complex patterns within vast datasets, generative models can generate biologically relevant structures or sequences that possess desired pharmacological activities, and combining these generative approaches with foundation models can further elevate their capabilities. Foundation models, such as large-scale GNNs and molecular transformers pretrained on diverse chemical and biological datasets, can encode a broader range of domain-specific knowledge and effectively capture generalizable molecular representations. Integrating generative models with foundation models improves their predictive power, stability, and generalization abilities, thus producing more meaningful and experimentally viable biomolecular designs with fewer computational resources^94^.

**Figure 6:**
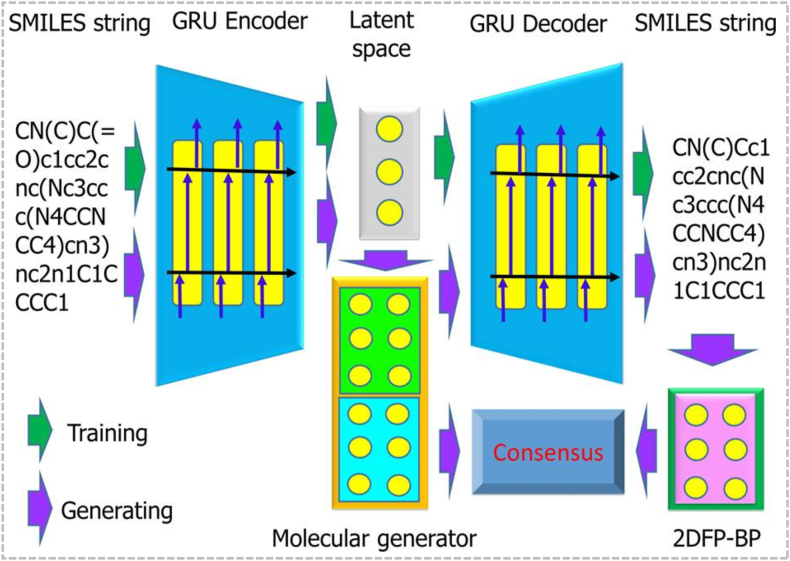
Schematic diagram of the generative network complex. First, the input SMILES string is encoded into the latent space. Then, the molecule generator optimizes the molecule in the latent space and reconstructs it into a new SMILES string through the decoder. Finally, the properties or activities of the generated molecules are evaluated by consensus, utilizing modules such as 2DFP-BP, thereby achieving a complete closed loop from training to molecule generation. The green arrows indicate the training process, while the purple arrows indicate the generation process^95^.

The GNC method proposed by Gao et al.^95^ in 2020 employed autoencoders and deep neural networks to optimize multiple chemical properties through gradient descent to automatically generate optimized drug molecules. This method has generated more than 1000 new molecules and provided alternative molecules that were superior to the original drugs for 8 marketed drugs (such as Ceritinib and Ribociclib), which has wide applications in small molecule drug design, MOO, and personalized drug discovery. Its schematic illustration is given in Fig. 6.

In the future, generative models built up on domain-specific foundation models are expected to become even more sophisticated and integral to the drug discovery pipeline. The main directions include combining more powerful foundation models (such as molecular transformers or graph-based transformer architectures) to improve the molecular characterization ability and the smoothness of the generation process, combining experimental data to optimize the actual feasibility of molecules, developing efficient MOO and improving generation algorithms (such as explainable AI models, quantum computing acceleration)^96^. Additionally, the conversion of peptides into small molecules also provides a feasible path for generative model-driven molecular design to convert candidate molecules into synthesizable and administrable drugs^97^. These advances will promote computer-aided drug design based on technologies such as GNC, assist in new-drug development and precision medicine, and further expand the application potential of AI in drug discovery.

### 5.3 Expanding the role of generative models in multi-modal biomolecular sciences

Generative models play a key role in multi-modal biomolecular sciences by integrating diverse data types, such as molecular structures, chemical properties, and phenotypic data. These models capture relationships between data sources, facilitating data fusion and overcoming the limitations of single-modal data^98^. In drug discovery and protein design, they optimize properties like drug efficacy or protein stability while generating novel molecular structures. They also enable cross-modal learning for better predictions of drug potency, safety, and efficacy^99^. Additionally, generative models help fill gaps in datasets by predicting missing data, such as chemical properties or experimental outcomes. These capabilities accelerate discovery, improve predictive accuracy, and drive innovation in biomolecular sciences, aiding drug development and molecular optimization^100^.

However, their application faces challenges, such as the heterogeneity of multi-modal data, which complicates unified representation and processing, affecting model performance^101^. Capturing complex, nonlinear relationships between modalities requires models to integrate data while preserving important features, demanding advanced model design and training^102^. The quality and completeness of multi-modal data, often noisy or imbalanced, also affect model generalization, and high-quality annotated datasets are scarce in biomolecular sciences. Complex architectures for data integration increase computational costs and may lead to training instability. Furthermore, ensuring the reliability and interpretability of generated outputs, especially in MOO, adds to the challenges. Lastly, the high computational demands of training multi-modal generative models limit their accessibility to resource-limited labs and institutions.

Future progress should focus on building high-quality multi-modal datasets, innovating model architectures, and ensuring reliable validation of results^103^. Developing lightweight models for resource-constrained environments will expand accessibility, accelerating breakthroughs in drug development, protein science, and precision medicine.

### 5.4 Multi-objective generation

Multi-objective generation is a method in drug design that optimizes multiple molecular properties simul-taneously, balancing factors such as multi-target BA, pharmacokinetics, toxicological properties, ADMET, and molecular novelty and synthesizability. Recently, the integration of generative models has advanced MOO, enabling efficient chemical space exploration and automated generation of potential drug molecules that meet multiple constraints^104^. Unlike traditional drug discovery methods, generative models driven by MOO use deep learning to analyze existing data distributions, ensuring the chemical validity of generated compounds while speeding up the process of creating and optimizing high-quality drug candidates. This approach significantly improves the efficiency and success rates of drug development.

**Figure 7:**
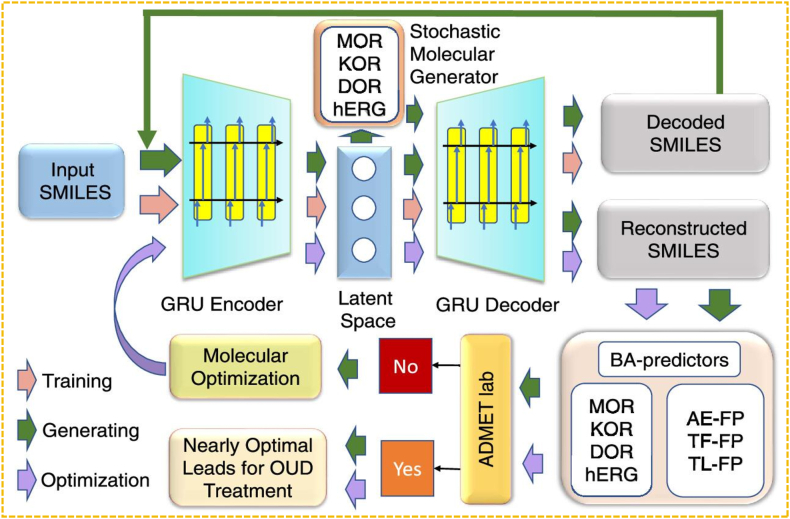
Schematic illustration of multitarget stochastic generative network complex, used to design novel compounds for the treatment of opioid use disorder (OUD). Three different paths, *i*.*e*., model training, molecular generation, and lead optimization are colored in pink, green, and purple, respectively. The pink path represents the model training process of the pretrained autoencoder network. The blue path signifies the compound generation process: the SMILES string of a given compound undergoes encoding in the encoding network, and its latent space representation is then fed into the stochastic molecular generator. The generated compounds are subsequently processed through the decoding–encoding network. The resulting molecules that have stable SMILESs are evaluated for their BA and ADMET (absorption, distribution, metabolism, excretion, and toxicity) properties. Molecules exhibiting desired BA and ADMET properties are regarded as nearly optimal leads. In cases where the properties are not satisfactory, molecular optimization (the purple path) is performed to generate more potential druglike compounds^99^.

In the field of molecular generation, Feng et al.^99^ proposed a multi-target stochastic generation network complex to optimize molecules targeting opioid receptors (MOR, KOR, DOR). This method combined GNC with MOO, integrating stochastic differential equations and autoencoder-based latent space optimization to enhance molecular diversity. It balanced BA, ADMET, and molecular novelty, which ensured high activity, favorable pharmacokinetics, and innovative structures. Key innovations included latent space optimization with DMs, using Langevin equations for molecular evolution, and employing pretrained autoencoders for filtering unstable candidates. Multi-step optimization improved drug activity and drug-likeness, offering new insights for opioid drug design. The schematic diagram of this multi-target stochastically generated network complex is presented in Fig. 7.

Future multi-objective generation in drug discovery will advance by integrating GNNs with RL to enhance model robustness-capturing molecular topology more accurately and dynamically optimizing generation strategies^105^. Additionally, the multi-target stochastic generation network complex method is expected to extend to drug design for neurodegenerative diseases, cancer, and infectious diseases, aligning with the trend toward polypharmacology. Improved ADMET predictions through self-supervised learning and high-throughput virtual screening will further accelerate lead compound selection and optimization, ultimately speeding up new drug development^106^.

## 6 Conclusions

Generative models are expected to revolutionize biomolecular sciences by offering innovative strategies for designing, predicting, and optimizing molecular structures in biochemistry, molecular biology, and protein science^107^. By generating new molecular entities from learned representations, these models hold significant promise in drug discovery, reaction prediction, protein structure design, and the development of molecules with customized properties. They simplify the discovery process and minimize dependence on conventional trial-and-error approaches, facilitating a more efficient exploration of extensive chemical and biological spaces.

Despite these advancements, challenges in data quality and model interpretability must be addressed to ensure the responsible use of generative models in molecular research^9^. Integrating models such as VAEs, GANs, and DMs with techniques such as quantum chemistry, MD, and RL can improve prediction accuracy.

Hybrid and multi-modal models, combined with experimental feedback, will further improve the practical utility of generative models in molecular challenges^68^.

Future trends in generative modeling will increasingly draw on automated generation using LLMs to autonomously encode chemical and biological knowledge, enabling simplified molecular discovery workflows. Simultaneously, multi-objective generative approaches will gain prominence, facilitating the concurrent optimization of multiple molecular properties, thus efficiently navigating complex chemical spaces for practical applications in drug discovery, bioengineering, and advanced protein design. Another trend is to utilize advanced mathematics, such as algebraic topology, combinatorial theory, and differential geometry, to regulate molecular generation and enhance structure prediction^46^.

By overcoming current limitations and using advances in ML and computational biology, these models can drive progress in personalized medicine, synthetic biology, and sustainable protein design, ultimately reshaping biomolecular sciences and accelerating innovation in medicine, material, and biology science^9^.

This review highlights the growing importance of generative models and calls for interdisciplinary collaboration to address challenges such as data quality and model interpretability, unlocking their full potential in advancing drug discovery, protein science, and synthetic biology.

## Author contributions

Jian Jiang was responsible for study design, manuscript writing, reviewing, and editing. Daixin Li and Guilin Wang collected relevant literature and prepared the initial draft. Nicole Hayes, Yazhou Shi, Huahai Qiu, Bengong Zhang reviewed and edited the manuscript. Tianshou Zhou, and GuoWei Wei supervised, conceptualized the manuscript, and revised the manuscript.

## Conflicts of interest

The authors declare no conflict of interest.
